# Current situation and influencing factors of economic exploitation of the older adult

**DOI:** 10.3389/fpubh.2025.1629960

**Published:** 2025-11-18

**Authors:** Kai Li

**Affiliations:** School of Finance and Taxation, Henan Finance University, Zhengzhou, China

**Keywords:** older adult, economic exploitation, MMSE, OAFEM, ADL, APGAR, PSSS

## Abstract

**Introduction:**

Against the backdrop of deepening population aging, the issue of economic exploitation among the older adult has become increasingly prominent, severely undermining their financial security and physical and mental well-being. This study aims to investigate the current status of economic exploitation among the older adult, analyze influencing factors such as cognitive function, social support, daily living abilities, and family care, and provide empirical evidence to improve the protection system for the older adults’ rights.

**Methods:**

A cross-sectional survey design was employed, selecting 455 older adult individuals from a certain province as the study subjects. Relevant variables were measured using the Mini-Mental State Examination, the Perceived Social Support Scale, the Activities of Daily Living Scale, and the Family Care Index Scale. Statistical analysis methods were used to identify key influencing factors of economic exploitation.

**Results:**

The survey revealed that the mean score for the Mini-Mental State Examination was 24.40, the mean score for the Perceived Social Support Scale was 53.89, the mean score for the Activities of Daily Living Scale was 32.50, and the mean score for the Family Care Index Scale was 7.89 (*p* < 0.001*). Older adult individuals in rural areas, with lower education levels, and limited financial resources were at higher risk of economic exploitation.

**Discussion:**

The occurrence of economic exploitation among the older adult is influenced by various factors, including cognitive function, social support, daily living abilities, and family care. To mitigate the risk of economic exploitation, the following measures are recommended: improving cognitive function in the older adult, particularly attention and computational abilities; strengthening the development of family and social support networks; enhancing daily living skills and providing assistance with financial matters; and increasing family care, especially by improving family members’ maturity in handling older adult-related issues. This research provides empirical evidence for formulating strategies to prevent economic exploitation among the older adult, contributing to safeguarding their financial security and improving their quality of life.

## 1 Introduction

With the acceleration of global population aging, the proportion of the older adult as a group in society is growing (1). 264.02 million people, or 18.70% of the population, are 60 years of age or older, according to data from China’s seventh national population census. The percentage of the population that is 60 years of age or older rose by 5.44 percentage points in comparison to 2010 (2). Social attention now centers on the well-being and quality of life (QOF) of the older adult. One of the most important factors in guaranteeing the QOF of the older adult is economic security. However, the percentage of the old population in society is rising as a result of the deepening of population aging, and the different issues they encounter have steadily gained attention (3). Among them, the problem of economic exploitation of the older adult is of particular concern. Economic exploitation not only directly harms the economic interests of the older adult, but also may have far-reaching negative effects on their physical and mental health, QOF, and social relationships. It can seriously threaten the well-being of older adult in their later life (4, 5).

In today’s society, the phenomenon of economic exploitation is becoming increasingly severe, which is heartbreaking. The consequences of economic exploitation not only lead to income inequality, but also directly affect the overall stability of society. Large groups of impoverished and exploited people often feel marginalized and helpless. This sense of social exclusion can easily lead to dissatisfaction and conflict. As a special vulnerable group in society, the older adult have significant group specificity in their disadvantaged position in economic affairs. Due to multiple factors such as natural decline in function, weakened cognitive judgment ability, and insufficient social participation, older adult are generally in a relatively disadvantaged position in terms of autonomous decision-making, risk avoidance, and rights protection in economic affairs. The economic exploitation of the older adult mainly falls into two categories. One is the economic abuse by relatives and caregivers. They exploit older adult by taking advantage of their emotional dependence or living attachment. They do this by forcibly demanding money, misappropriating savings and pensions, privately selling assets, and overcharging or deducting expenses. This exploitation is covert and persistent. The second type is fraud by others. Those without direct and close relationships can fabricate facts and take advantage of the older adults’ weak cognition and poor prevention to defraud money through false investment, identity impersonation, and telecommunications and online fraud. They are highly deceptive and can easily target the psychological needs of the older adult to succeed (6). In 2024, procuratorial organs across the country approved the arrest of over 200 individuals in more than 310 cases involving fraud targeting older adult, and prosecuted over 630 individuals in more than 1,700 cases for related crimes.

Current research on the economic exploitation of the older adult mainly uses the Chinese-English version of the Older Adult Financial Exploitation Measure (OAFEM) and the Mini-Mental State Examination (MMSE) as assessment tools. The OAFEM scale has been widely used to assess the phenomenon of older adult financial exploitation. The MMSE scale, on the other hand, is mainly used to assess the cognitive function of older adults to determine whether they are more vulnerable to financial exploitation due to cognitive impairment (7, 8). In a related study, Herrenkohl et al. (9) provided an overview of the life cycle theory of violence. The study utilized the OAFEM scale to point out the association of older adult abuse with childhood abuse, adolescent violence, and intimate partner violence (9). Using the OAFEM scale, Dahlberg et al. (10) found that loneliness was a significant risk factor for mental health in older adults and could be related to psychological trauma following economic exploitation. Regarding the MMSE scale, Gallegos et al. (11) reviewed its 45 years of development and pointed out that the scale was valuable in assessing cognitive functioning in older adults, but there were problems with cultural adaptation and language differences. Truong et al. (12) further validated the validity of the MMSE scale and its dimensions through network analysis, emphasizing its wide application in clinical and research settings. Research on the economic exploitation of the older adult is still lacking, though, and more needs to be done to fully comprehend the issue’s current state and thoroughly examine the variables that contribute to it (13). Only by fully recognizing the seriousness and complexity of the economic exploitation of older persons and by thoroughly analyzing the multiple influencing factors behind it can effective preventive and intervention measures be formulated. In this way, the economic rights and interests of the older adult can be effectively protected, and a safe, harmonious and dignified living environment in old age can be created for them. In view of this, the study intends to adopt the questionnaire survey method to explore the current situation of economic exploitation of the older adult and its influencing factors. Through this study, it aims to provide scientific references for government departments to formulate relevant policies and regulations, social organizations to provide targeted services, and family members to enhance their awareness of prevention. In this way, the legitimate rights and interests of the older adult may be effectively protected, a complete system for the protection of their economic rights and interests can be built, and the fairness, justice, and peaceful growth of society can be encouraged.

## 2 Materials and methods

### 2.1 Research target

#### 2.1.1 Inclusion criteria

Eligible participants are older adults aged 60 years and older who have lived in the study area for at least 1 year. They must have the cognitive ability to clearly recall and describe their experiences with economic exploitation and related situations. Participants must also agree to complete the informed consent form and take part in the study.

#### 2.1.2 Exclusion criteria

Older adults with severe cognitive impairment that prevents them from accurately recalling and describing events and older adults with severe communication disorders that prevent them from communicating effectively.

#### 2.1.3 Elimination criteria

Samples are found to have too much missing key information. In cases where duplicate samples of the same older adults are verified and found to be included in the study sample, the duplicate samples are excluded and only one valid data copy is retained. After data review, it is found that there are obvious anomalies or logical errors in the survey data of some of the older adults, and those that cannot be corrected through further verification. A total of 480 questionnaires are distributed in the study and 455 questionnaires are valid, with a validity rate of 94.79%.

#### 2.1.4 Sample size estimation

The research survey tools include 16 general demographic data items, 5 dimensions of MMSE scale, 6 dimensions of the Chinese English version of the older adult Economic Exploitation Scale, 3 dimensions of the Daily Living Ability Scale, 5 dimensions of the Family Care Index Scale, and 4 dimensions of the Perceived Social Support Scale (PSSS), totaling 39 items. The basic sample size is calculated using the principle of designing a sample size that is 10 times the dimensionality of the scale in academic research. This results in a sample size of 390 cases, with 39 dimensions multiplied by 10. At the same time, considering the possibility of invalid samples during the investigation process, a 20% sample redundancy is reserved. Therefore, the sample size to be distributed is 487.5. As the sample size needs to be an integer, 488 questionnaires will be distributed in the end. Therefore, a total of 488 questionnaires are distributed in the study, with 455 valid questionnaires and a questionnaire effectiveness rate of 93.24%.

#### 2.1.5 Sampling method

The study conducts a convenience sampling method from August to October 2024, selecting older adult residents in a certain province who meet the inclusion and exclusion criteria in the community.

### 2.2 Research methods

#### 2.2.1 General information questionnaire

As indicated in Table 1, the study creates a general information questionnaire for the older adult based on its objectives.

**Table 1:** General data of the older adult (*n* = 455).

Variable	Category	Frequency	Composition ratio (%)
Gender	Male	241	52.97
	Female	214	47.03
Place of residence	Village	207	45.49
	Towns	248	54.51
Age	60 ~ 70	159	34.95
	71 ~ 80	216	47.47
	>80	80	17.58
Educational level	Never went to school	140	30.77
	Primary school	118	25.93
	Junior high school	123	27.03
	Technical school, high school	65	14.29
	College or above	9	1.98
Marital status	Be married	299	65.71
	Divorce	44	9.67
	Widowed	112	24.62
Household	Live alone	53	11.65
	Live only with his wife	206	45.27
	Live with children’s families only	72	15.82
	Live with wife and children’s family	65	14.29
	Live with other relatives	53	11.65
	Live with a babysitter or caregiver	6	1.32
Number of children	1	61	13.41
	2	113	24.84
	3	165	36.26
	≥4	116	25.49
Frequency of child visits	Several times a year	155	34.07
	Several times a month	85	18.68
	Several times a week	110	24.18
	Everyday	105	23.07
Ways of visiting children	Visit at home	222	48.79
	Phone or video	233	51.21
Community visit frequency	Never	230	50.55
	Seldom	127	27.91
	At times	78	17.14
	Frequently	20	4.40
Pre-retirement occupation	Business, service personnel	61	13.41
	Agriculture, forestry, animal husbandry, fishing, water conservancy production personnel	185	40.66
	Heads of state organs, party and mass organizations, enterprises and public institutions	24	5.27
	Professional technical personnel	63	13.85
	Clerical and related personnel	42	9.23
	Production, transportation equipment operators, and related personnel	80	17.58
Economic source	Self-employment income	58	12.75
	Pension/pension	132	29.01
	Spouse support	71	15.60
	Child supply	140	30.77
	Government welfare subsidy	54	11.87
Monthly income	<500	208	45.71
	500 ~ 1,600	91	20.00
	500 ~ 1,600	132	29.01
	≥5,000	24	5.28
Income custodian	Oneself	328	72.09
	Mate	86	18.90
	Sons and daughters	31	6.81
	Other	10	2.20
Number of chronic diseases	No	174	38.24
	1 or more kinds	258	56.70
Medical security	There is no medical insurance	130	28.57
	Employee medical insurance	93	20.44
	Medical insurance for urban and rural residents	232	50.99

#### 2.2.2 Research tools

The MMSE is one of the most influential screening tools for cognitive deficits at home and abroad. The screening content of this scale mainly includes orientation, memory, calculation, language, visuospatial, utilization and attention (14). Higher scores indicate greater cognitive functioning of the patient (15). The scale is rated out of a total of 30 points. The OAFEM is an instrument used to assess whether older adults are economically exploited. The scale is developed to detect, by means of self-report, whether an older person is ever subjected to unlawful or inappropriate use of his or her funds, property, or resources, as well as the interests of others (16). The OAFEM covered six main domains, which are theft and fraud, trust abuse, financial entitlement, coercion, possible signs of financial abuse, and money management difficulties (17). The Activities of Daily Living Scale (ADL) adopted in the research is developed and formulated by American scholars Lawton and Brody in 1969 (18). This scale is composed of two dimensions: the Physical Self-care Scale and the Instrumental ADL. It includes six aspects related to self-care and eight aspects related to the ability to use tools. Those related to self-care in daily life include eating, dressing, grooming, using the toilet, walking and bathing. The ability to use tools encompasses a wide range of activities, such as making phone calls, shopping, doing housework, washing clothes, walking, using transportation, taking medicine, and managing finances independently. Higher scores indicated a greater capacity for self-care; the total score varied from 0 to 14 (19). The Family APGAR Index (APGAR) was an instrument used to assess family functioning, designed and first presented by Smilkstein (20) of the University of Washington, Seattle, USA. The APGAR scale consists of five dimensions: fitness, cooperation, growth, emotion, and intimacy. Each dimension contains one item and uses the 3-level Likert scoring method. The score range is 0, 1, and 2, with a total possible score of 0 to 10 points (21, 22). The PSSS is a tool designed to examine the level of perceived social support (PSS) of an individual. Zimet et al. (23) created the scale in 1988, and it has since been extensively utilized in numerous investigations. The PSSS used a 7-point Likert scale to rate each of the 12 entries that assessed an individual’s perceived level of social support from friends, family, and other significant others, ranging from “Strongly Disagree” to “Strongly agree.” Higher scores indicated stronger felt social support, with total scores ranging from 12 to 84 (24, 25). All relevant survey forms will be consolidated into a comprehensive questionnaire on the health and economic status of older adults. This questionnaire will be used to collect relevant information from older adults within the study area between August and October 2024.

### 2.3 Statistical analysis

Data are collected through questionnaires, and SPSS software is used for data entry and statistical analysis. Descriptive statistics are used to analyze the scores of each scale. One-way ANOVA and correlation analysis are used to explore the relationship between the factors and economic exploitation. Multiple linear regression analysis is further used to determine the main factors affecting the economic exploitation of the older adult.

### 2.4 Ethical principles

The principle of voluntariness. There is a need to ensure that the participation of all research subjects is completely voluntary and free from any form of coercion or inducement. Participants are free to leave the study at any moment without facing any consequences.

The principle of confidentiality. The anonymity of all data is ensured during questionnaire design and data collection. No personally identifiable information, such as name, identity card number, etc., is recorded on the questionnaire.

The principle of do no harm. The potential risks to the research subjects are minimized during the design and implementation of the study. The content of the questionnaire and the survey process should avoid causing discomfort or psychological stress to the study participants.

## 3 Results

### 3.1 Reliability and validity test

To ensure the matching degree and scientific nature of the items of the scale used in the research with the research topic, the research adopts a combination of on-site questionnaire distribution and email invitation. This is done to select 10 experts. The experts has professional backgrounds in geriatrics, social work, clinical psychology and scale development. They are invited to evaluate the content validity of each item of the scale involved in the research. To quantitatively test the content validity, two internationally recognized evaluation indicators, item content validity index (I-CVI), are introduced. With 10 experts, I-CVI is greater than 0.78. This indicates that the scale items fit well with the research purpose and have good content validity. They can be used for subsequent formal investigations.

Cronbach’s *α* is a measure of the reliability of a scale or test. It solves the shortcomings of the partial folding approach and is the most often employed method of reliability analysis in social science research (26). Therefore, the study adopts Cronbach’s *α* method to test the reliability of each scale. Table 2 displays the test results.

**Table 2:** Reliability test of each scale.

/	Cronbach’s α value	Reliability result
APGAR	0.930	Good
MMSE	0.882	Good
ADL	0.930	Good
OAFEM	0.837	Good
PSSS	0.886	Good

In Table 2, the reliability of the scales is high overall, with Cronbach’s *α* values above 0.8 for all scales. It indicates that the reliability of these scales are all at a good level and can measure the corresponding concepts or traits more stably and reliably.

### 3.2 Examination of the outcomes of the existing state of older adult economic exploitation

To understand the current status of economic exploitation of the older adult, the study analyzes the status of economic exploitation scores of the older adult, summary mental status examination scores, PSS scores, ADL scores, and family care scores of the older adult. The economic exploitation score of the older adult is shown in Table 3.

**Table 3:** Economic exploitation scores of older persons.

Item	Score range	Scoring range	Dimension score	Entry equalization	Sort
Economic exploitation	0 ~ 60	0 ~ 20	8.45 ± 5.23	0.28 ± 0.17	/
Theft and fraud	0 ~ 12	0 ~ 6	2.13 ± 1.45	0.35 ± 0.24	1
Abuse of trust	0 ~ 18	0 ~ 9	3.27 ± 2.10	0.36 ± 0.23	2
Financial right	0 ~ 6	0 ~ 3	1.24 ± 0.86	0.40 ± 0.28	3
Coerce	0 ~ 18	0 ~ 8	1.51 ± 1.03	0.25 ± 0.15	4
Possible abusive tendencies	0 ~ 12	0 ~ 5	0.46 ± 0.32	0.08 ± 0.06	5

**Figure 1:**
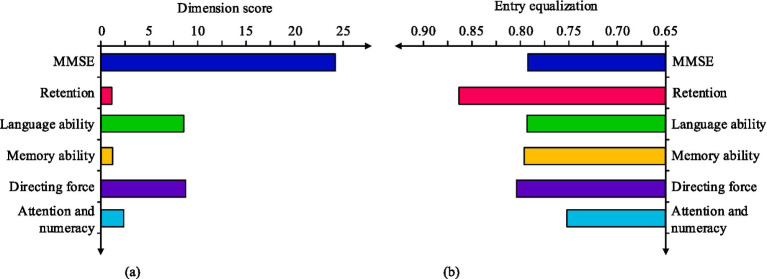
Scores of the simple mental state examination of the older adult. **(a)** The score of older adults’ simple mental state examination dimension. **(b)** The dimensionality items of simple mental state examination for the older adult were equally divided.

In Table 3, economic exploitation has a total score between 0 and 60. The older adult have a mean score of 8.45 ± 5.23 and an entry mean score of 0.28 ± 0.17, with scores ranging from 0 to 20. There are some differences in the mean scores of the entries for each dimension. In descending order, they are financial right, abuse of trust, theft and fraud, coerce, and possible abusive tendencies. The scores of simple mental state examination of the older adult examination are shown in Figure 1.

In Figure 1a, the mean of the total MMSE score is 24.40 with a standard deviation of 0.13. In Figure 1b, the highest mean score is found for the entries on retention, which is 0.88 ± 0.15. The lowest mean score is found for the entries on attention and numeracy, which is 0.75 ± 0.22. Scores of PSS of the older adult in various dimensions are shown in Table 4.

**Table 4:** Scores of perceived social support of the older adult in various dimensions.

Item	Score range	Scoring range	Dimension score	Entry equalization	Sort
Perceived social support	12 ~ 84	14 ~ 82	53.89 ± 15.20	4.49 ± 1.28	/
Family support	4 ~ 28	4 ~ 28	18.45 ± 5.12	4.59 ± 1.31	1
Friend support	4 ~ 28	3 ~ 28	18.16 ± 5.39	4.50 ± 1.38	2
Other support	4 ~ 28	4 ~ 28	17.39 ± 5.75	4.37 ± 1.38	3

In Table 4, the mean total score of PSS of the older adult is 53.89 and the mean score of entries is 4.49. Family support is the main source of PSS of the older adult with the highest score of 18.45 ± 5.1. Friend support scores a higher high as 18.16 ± 5.39 and the mean score of entries is 4.50 ± 1.38. Other support has the lowest score of 17.39 ± 5.75 and the mean score of entries is 4.37 ± 1.38. Table 5 displays the ADL scores for the older adult.

**Table 5:** Scores of ADL of the older adult.

Item	Score range	Scoring range	Dimension score	Entry equalization	Sort
ADL	0 ~ 80	10 ~ 60	32.50 ± 10.20	1.63 ± 0.51	/
Instrumental daily living ability	0 ~ 48	6 ~ 36	19.70 ± 4.80	1.64 ± 0.40	1
Basic ability of daily living	0 ~ 32	4 ~ 24	12.80 ± 5.60	1.60 ± 0.45	2

In Table 5, the mean score of ADL is 32.50 ± 10.20, and the mean score of entries is 1.63 ± 0.51, which puts the overall ADL of older adults at a moderate level. The mean score of basic ADL is 12.80 ± 5.60, and the mean score of the entries is 1.60 ± 0.45. The mean score of instrumental ADL is 19.70 ± 4.80, and the mean score of the entries is 1.64 ± 0.40. However, from the overall point of view, the scores of the older adults’ ADL scores are generally not high, indicating that older adults have some difficulty in accomplishing daily living activities. Table 6 displays the older individuals’ family care scores.

**Table 6:** Family care scores of the older adult.

Item	Score range	Scoring range	Dimension score	Entry equalization	Sort
Family caring degree	0 ~ 10	0 ~ 10	7.89 ± 0.23	1.58 ± 0.05	/
Intimacy	0 ~ 2	0 ~ 2	1.89 ± 0.32	0.95 ± 0.16	1
Affective degree	0 ~ 2	0 ~ 2	1.67 ± 0.41	0.84 ± 0.21	2
Fitness	0 ~ 2	0 ~ 2	1.56 ± 0.45	0.78 ± 0.23	3
Cooperation degree	0 ~ 2	0 ~ 2	1.45 ± 0.36	0.73 ± 0.18	4
Growth degree	0 ~ 2	0 ~ 2	1.32 ± 0.54	0.66 ± 0.27	5

In Table 6, the mean score of overall family care status is 7.89 ± 0.23 and the mean score of entries is 1.58 ± 0.05. It indicates that the overall level of family care among the older adult is high. The highest mean score of intimacy is 1.89 ± 0.32, and the mean score of the entries is 0.95 ± 0.16. It indicates that intimacy between older adults and family members is the core of family care. The mean score for growth degree is the lowest at 1.32 ± 0.54, and the mean score of the entries is 0.66 ± 0.27. This indicates that the intimacy and independence of family members in dealing with older adult need to be further improved.

### 3.3 Analysis of factors affecting the economic exploitation of older persons

The effects of gender, residence, and age on economic exploitation of the older adult is shown in Table 7.

**Table 7:** Effects of gender, residence, and age on economic exploitation of the older adult (*n* = 455).

Variable	Category	The number of people without FE (%)	The number of people with FE (%)	*χ* ^2^	*P*
Gender	Male	62 (25.7%)	179 (74.3%)	1.823	0.182
	Female	50 (23.4%)	164 (76.6%)		
Place of Residence	Village	44 (21.2%)	163 (78.8%)	5.785	0.015*
	Towns	85 (34.3%)	163 (65.7%)		
Age	60 ~ 70	53 (33.3%)	106 (66.7%)	1.786	0.410
	71 ~ 80	99 (45.8%)	117 (54.2%)		
	>80	21 (26.3%)	59 (73.8%)		

*p < 0.05.

In Table 7, gender has no discernible impact on the prevalence of older adult economic exploitation, *p* > 0.05. There is no significant difference (SD) in the risk of economic exploitation between male and female older persons. The prevalence of older adult economic exploitation is significantly influenced by one’s place of residence (*p* < 0.05), with rural older persons being at a significantly higher risk of economic exploitation than urban older persons. Age has no significant effect on the incidence of economic exploitation of older persons, *p* > 0.05. The risk of economic exploitation does not differ for older adults of different ages. Table 8 displays the findings of the relationship between literacy and marital status and the economic exploitation of the older adult.

**Table 8:** Effects of education level and marital status on economic exploitation of the older adult (*n* = 455).

Variable	Category	The number of people without FE (%)	The number of people with FE (%)	*χ* ^2^	*P*
Educational level	Never went to school	46 (32.9%)	94 (67.1%)	21.114	<0.001*
	Primary school	28 (23.7%)	90 (76.3%)		
	Junior high school	33 (26.8%)	90 (73.2%)		
	High school, technical school, technical school	19 (29.2%)	46 (70.8%)		
	College or above	6 (66.7%)	3 (33.3%)		
Marital status	Be married	116 (38.8%)	183 (61.2%)	5.083	0.062
	Divorce	3 (6.8%)	41 (93.2%)		
	Widowed	27 (24.1%)	85 (75.9%)		

*p < 0.05.

In Table 8, the chi-square test result of literacy on the economic exploitation of the older adult is 21.114, *p* < 0.001. It demonstrates that the prevalence of economic exploitation of the older adult is highly influenced by literacy, and that the risk of economic exploitation is much higher for older adult with lower literacy levels than for those with higher literacy levels. The chi-square test result of marital status on economic exploitation of the older adult is 5.083, *p* = 0.062. It shows that marital status has no significant effect on the occurrence of economic exploitation of the older adult. The results of the effect of household and number of children on the economic exploitation of the older adult are shown in Table 9.

**Table 9:** Effects of residence style and number of children on economic exploitation of the older adult (*n* = 455).

Variable	Category	The number of people without FE (%)	The number of people with FE (%)	*χ* ^2^	*P*
Household	Live alone	23 (43.4%)	30 (56.6%)	4.845	0.428
	Live only with his wife	74 (35.9%)	132 (64.1%)		
	Live with children’s families only	32 (44.4%)	40 (55.6%)		
	Live with wife and children’s family	30 (46.2%)	35 (53.8%)		
	Live with other relatives	25 (47.2%)	28 (52.8%)		
	Live with a babysitter or caregiver	3 (50.0%)	3 (50.0%)		
Number of children	1	47 (77.0%)	14 (23.0%)	6.076	0.112
	2	52 (46.0%)	61 (54.0%)		
	3	58 (35.2%)	107 (64.8%)		
	≥4	53 (45.7%)	63 (54.3%)		

*p < 0.05.

**Figure 2:**
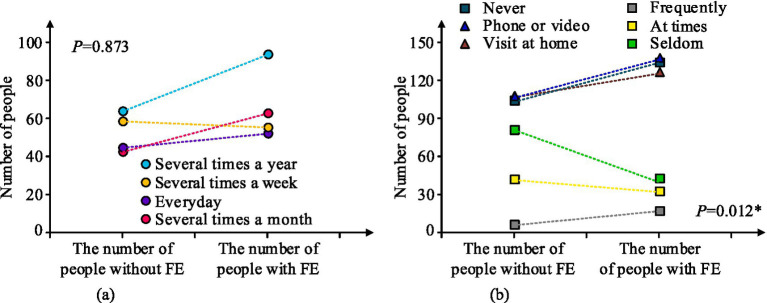
Effects of frequency, mode, and community visit frequency of children on economic exploitation of the older adult. **(a)** The impact of child visitation frequency on economic exploitation of older adults. **(b)** The impact of child visitation methods and community visitation frequency on economic exploitation of older adults.

The statistics in Table 9 shows that the chi-square value of household is 4.845 with a *p* = 0.428. It shows that there is no significant effect of household on the incidence of economic exploitation of the older adult. The chi-square value of number of children is 6.076 with *p* = 0.112. It demonstrates that the number of children has no discernible impact on the prevalence of older adult economic exploitation. The outcomes of the effect of the frequency and style of children’s visits and the frequency of community visits on the economic exploitation of the older adult are shown in Figure 2.

### 3.4 Children on economic exploitation of the older adult

In Figure 2a, the incidence of economic exploitation of older adult is not significantly impacted by the frequency of child visitation, while a somewhat larger percentage of older adult who visit their children less frequently are exploited economically, *p* > 0.05. In Figure 2b, there is no significant effect of child visitation mode on the incidence of economic exploitation of the older adult, *p* = 0.873. There is a significant effect of community visitation frequency on the incidence of economic exploitation of the older adult, *p* = 0.012*. Older adults with lower frequency of community visitation are at significantly higher risk of financial exploitation than those with higher frequency of community visitation. The results of the effect of pre-retirement occupation and source of livelihood on the economic exploitation of older persons are shown in Table 10.

**Table 10:** Effects of pre-retirement occupation and source of income on economic exploitation of the older adult (*n* = 455).

Variable	Category	The number of people without FE (%)	The number of people with FE (%)	*χ* ^2^	*P*
Pre-retirement occupation	Heads of state organs, party and mass organizations, enterprises and public institutions	8 (33.3%)	16 (66.7%)	14.095	0.008*
	Professional technical personnel	51 (81.0%)	12 (19.0%)		
	Clerical and related personnel	32 (76.2%)	10 (23.8%)		
	Business, service personnel	46 (75.4%)	15 (24.6%)		
	Agriculture, forestry, animal husbandry, fishing, water conservancy production personnel	78 (42.2%)	107 (57.8%)		
	Production, transportation equipment operators and related personnel	55 (68.8%)	25 (31.3%)		
Economic source	Self-employment income	43 (74.1%)	15 (25.9%)	34.151	0.001*
	Pension/pension	110 (83.3%)	22 (16.7%)		
	Spouse support	59 (83.1%)	12 (16.9%)		
	Child supply	112 (80.0%)	28 (20.0%)		
	Government welfare subsidy	45 (83.3%)	9 (16.7%)		

*p < 0.05.

In Table 10, in terms of pre-retirement occupation, the chi-square test result is 14.095, with a *p* = 0.008. It demonstrates that the prevalence of older adult economic exploitation and pre-retirement employment are correlated. Among them, the number of people in agriculture, forestry, animal husbandry, fishery, and water conservancy production who have economic exploitation is relatively high, which may be related to some characteristics of this occupational group in economic and social aspects. Further investigation may be necessary to determine the precise causes of the comparatively large percentage of individuals in charge of state organs, party organizations, businesses, and institutions who are economically exploited. In terms of economic sources, the result of the chi-square test is 34.151, *p* = 0.001. It displays that there is an extremely SD between economic sources and the occurrence of economic exploitation of the older adult. In this case, the relative incidence of economic exploitation is lower in the case of older persons whose economic source is their own income from work and pension or old age. The occurrence of economic exploitation also varies under other modes of economic sources. This may be related to factors such as stability and availability of different economic sources. Overall, both pre-retirement occupation and economic sources have a significant impact on the economic exploitation of the older adult. The results of the effects of monthly income and income custody on economic exploitation of the older adult are shown in Table 11.

**Table 11:** Effects of income and income custody on economic exploitation of the older adult (*n* = 455).

Variable	Category	The number of people without FE (%)	The number of people with FE (%)	*χ* ^2^	*P*
Monthly income	<500	93 (44.7%)	115 (55.3%)	9.873	0.016*
	500 ~ 1,600	22 (24.2%)	69 (75.8%)		
	500 ~ 1,600	60 (45.5%)	72 (54.5%)		
	≥5,000	14 (58.3%)	10 (41.7%)		
Income custodian	Oneself	146 (44.5%)	182 (55.5%)	3.670	0.219
	Mate	32 (37.2%)	54 (62.8%)		
	Sons and daughters	13 (41.9%)	18 (58.1%)		
	Other	5 (50.0%)	5 (50.0%)		

*p < 0.05.

In Table 11, the prevalence of financial exploitation among older adults is significantly influenced by monthly income *p* < 0.05. Older adults with less than 500 yuan and 500–1,600 yuan are at significantly higher risk of financial exploitation than those with ≥5,000 yuan. Income custodian has no significant effect on the occurrence of economic exploitation of the older adult, *p* = 0.219. Table 12 displays the findings of the relationship between the frequency of chronic diseases (CD) and the kind of medical insurance (MI) and the financial exploitation of the older adult.

**Table 12:** Effects of number of chronic diseases and type of medical insurance on economic exploitation of the older adult (*n* = 455).

Variable	Category	The number of people without FE (%)	The number of people with FE (%)	*χ* ^2^	*P*
Number of chronic diseases	No	75 (43.1%)	99 (56.9%)	0.215	0.645
	1 or more kinds	121 (43.1%)	160 (56.9%)		
Medical security	There is no medical insurance	47 (36.2%)	83 (63.8%)	9.783	0.007*
	Employee medical insurance	57 (61.3%)	36 (38.7%)		
	Medical insurance for urban and rural residents	89 (38.4%)	143 (61.6%)		

*p < 0.05.

In Table 12, the quantity of CDs has no discernible impact on the economic exploitation of the older adult, according to the *p* = 0.645 for the number of CDs. The chi-square test value for MI is 9.783 with a *p* = 0.007*. It indicates that there is a SD between the type of MI and the occurrence of economic exploitation of the older adult. In this case, the incidence of economic exploitation among the older adult without any MI is relatively high, while the incidence of economic exploitation among the older adult with employee MI is relatively low. This may indicate that the availability and type of health insurance affects the economic status and risk coping capacity of older persons, which in turn affects their likelihood of being financially exploited. The older adult who have better health insurance coverage are relatively less likely to be economically exploited. The results of the effects of ADL and family care on the economic exploitation of older persons are shown in Table 13.

**Table 13:** Effects of daily living ability and family caring degree on economic exploitation of the older adult (*n* = 455).

Variable	Category	The number of people without FE (%)	The number of people with FE (%)	*χ* ^2^	*P*
BADL Ability	Normal ability of daily life	88 (52.4%)	80 (47.6%)	5.126	0.024*
	Impaired ability to live daily	90 (37.9%)	147 (62.1%)		
IADL Ability	Normal ability of daily life	65 (60.2%)	43 (39.8%)	12.873	<0.001**
	Impaired ability to live daily	110 (32.1%)	234 (67.9%)		
Family caring degree	There is a serious lack of family care	4 (3.3%)	116 (96.7%)	86.746	<0.001*
	Moderate lack of family caring	12 (8.5%)	130 (91.5%)		
	The degree of family care is higher	147 (76.2%)	46 (23.8%)		

*p < 0.05.

In Table 13, BADL focuses on physiological self-care ability. Its impairment primarily reflects older adults’ dependence on physical care and has a relatively low direct correlation with FE-related scenarios, such as economic decision-making and asset management. Therefore, the increase in risk is limited. IADL includes modules directly related to economic activities such as financial management and shopping payments. Impairment means older adult lose the ability to control their finances independently and identify financial risks. They must rely on others to handle their economic affairs, which directly increases FE risks, such as the illegal occupation of assets and improper consumption. The results of the chi-square test of family care degree shows that *p* < 0.001. It indicates that family care degree is statistically significant to indicate that the lower the family care degree, the higher the risk of economic exploitation of the older adult. The characteristics that significantly influence older individuals’ financial exploitation are analyzed using binary logistic regression using SPSS 26.0. Since the χ^2^ is highest for the economic source of the older adult and the family care degree of the older adult, the study will analyze the logistic regression analysis (LRA) for these two independent variables. The results are shown in Table 14.

**Table 14:** LRA of factors affecting economic exploitation of the older adult (*n* = 455).

Variable	Item	Reference group	S. E	OR	*β*	*R* ^2^	95%CI	*P*
Economic source	Pension or pension	Self-employment income	1.125	0.053	−2.919	6.780	0.005 ~ 0.490	0.007*
	Child support		0.928	0.069	−2.665	8.203	0.010 ~ 0.450	0.005*
	Government welfare subsidies		1.700	0.011	−4.683	7.587	0.000 ~ 0.260	0.007*
Family caring degree	The degree of family care is higher	Court care is seriously lacking	1.360	0.003	−5.889	18.820	0.000 ~ 0.050	0.001*

*p < 0.05.

In Table 14, the regression coefficient related to the economic exploitation of the older adult with their own work income as their source of income in the effect of economic source on the economic exploitation of the older adult is 2.919, *p* = 0.007, which is a statistically SD, and the dominance ratio OR = 0.053, with a 95% confidence interval (CI) of 0.005 to 0.490. The regression coefficient related to the economic exploitation of the older adult with child support as the economic source is −2.665, *p* = 0.005, OR = 0.069, with a 95% CI of 0.010 to 0.450. This shows that the economic exploitation of older persons whose financial resources are based on child support is significantly lower than that of older persons whose livelihood is based on pensions or old-age pensions. The older adult who live on government related welfare benefits have a *p* = 0.007, *β* = −4.683, OR = 0.011, and a 95% CI of 0.000 to 0.260. This indicates that older persons who depend on government-related welfare benefits are significantly less likely to be economically exploited than those who depend on pensions or retirement benefits as a source of livelihood. In terms of family care degree, older adult with higher family care degree have a *p* = 0.001, *β* = −5.889, S. E = 1.360, OR = 0.003, and a 95% CI of 0.000 to 0.050, as compared to those with a severe lack of family care degree.

## 4 Discussion

The outcomes of the questionnaire survey indicated that the current status of economic exploitation of the older adult is characterized by multiple dimensions. The mean score of economic exploitation items was 0.28 ± 0.17, and the performance of each dimension was different. The financial right dimension scores were relatively high, and the possible abusive tendencies dimension scores were low, indicating that different types of economic exploitation behaviors differed significantly in specific aspects. Therefore, the prevention of economic exploitation should focus on financial right protection and explore potential risk factors such as irregular financial management of the family. On cognitive function, the mean value of MMSE was 24.40 with a standard deviation of 0.13. Most of the older adults had good cognitive function. Among them, memory was optimal, and attention and calculation needed to be focused. Regarding social support, the mean value of PSS of the older adult was 53.89, and the mean score of the entries was 4.49, which was at a medium-high level. Family support had the highest score, emphasizing the importance of family, but there were large individual differences in friend support. Enriching the social support network and enhancing the stability of friend support could provide the older adult with more avenues to seek help for financial problems and reduce the possibility of exploitation. The mean value of ADL for the older adult was 32.50 ± 10.20, and the mean entry score was 1.63 ± 0.51. The overall ADL was moderate, and both basic and instrumental ADL needed attention. Older persons with impaired ADL were more vulnerable to economic exploitation, as they were more dependent on others to manage their financial affairs (27). Providing social assistance to this group of older persons, such as adding financial assistance to home care, was essential to preventing financial exploitation. In terms of family care, the mean value of overall family care status was 7.89 ± 0.23, and the mean score of entries was 1.58 ± 0.05, which was high but the level of maturity needs to be improved. Family care degree was closely related to economic exploitation. The lower the care degree, the higher the risk of exploitation of the older adult, which was consistent with the findings of Boyle et al. (28). Families should enhance their maturity in dealing with the older adult, strengthen communication, rationally plan economic affairs, and create a caring atmosphere to reduce the risk of economic exploitation.

In terms of demographic characteristics, gender, age, marital status, household, and number of children had no significant effect on the incidence of economic exploitation of the older adult. However, place of residence had a significant effect. Due to disparities in economic development, social security system improvement, and social conceptions between urban and rural areas, older adult in rural areas were much more likely to be victimized by economic exploitation than their urban counterparts. Therefore, the rural social security system should be improved, and the government should support rural communities’ economic development. The economic exploitation of the older adult was significantly impacted by literacy. Older adults with low literacy level were difficult to recognize and prevent economic exploitation due to lack of financial knowledge and weak legal awareness. This result was similar to the findings of scholar Boeyink and scholar Falisse (29). Pre-retirement occupations and economic sources had a clear impact on the economic exploitation of the older adult. By strengthening the pension system and encouraging older adult to work as long as they are physically capable of doing so, the government could try to increase the stability of the older adults’ financial resources. The incidence of older adult economic exploitation was significantly influenced by monthly income. The government could help the older adult to improve their income and enhance their sense of economic security through policy support and employment guidance (30). Regarding MI, there was a SD between the type of MI and the prevalence of older adult economic exploitation. The Government could further expand the scope of MI coverage and raise the level of MI protection, especially to strengthen the protection of older persons without MI. In terms of the relationship between economic resources and economic exploitation, compared with pensioners, those who relied on their own income from work, children’s support, and government welfare subsidies were significantly less likely to be economically exploited, indicating the importance of stable economic resources to the economic security of the older adult. In terms of family care, the likelihood of financial exploitation was significantly lower among older adults with a higher degree of family care than among older adults with a severe lack of family care. It can be concluded that strengthening family care and ensuring the stability of financial resources for older persons were key measures to prevent economic exploitation.

In summary, the economic exploitation of older persons is affected by a combination of factors. To effectively reduce the phenomenon of economic exploitation of the older adult, it is necessary for the Government, society and families to make joint efforts. The Government should improve the social security system, strengthen the construction of rural areas, and raise the level of income and MI protection for the older adult. Society should carry out knowledge dissemination and education activities to enhance the ability of older persons to protect themselves. Families should raise the level of care and rationally plan the economic affairs of the older adult. Through coordinated interventions by various parties, the economic rights and interests of the older adult should be effectively safeguarded and their QOF improved.

## 5 Conclusion

The study revealed the current situation of financial exploitation of older adult and its main influencing factors by analyzing survey data from 455 older adult. The study found that the overall level of financial exploitation of older adults was low, but the financial right dimension scored high, indicating that implicit financial control and trust abuse were the main manifestations of financial exploitation. First, the stability of social support networks played an important role in preventing economic exploitation. Family support scored the highest, but individual differences in friend support were significant, suggesting the need to enhance the diversity of social support networks. Second, older adults with impaired ADL were more likely to be economically exploited, *p* = 0.006*, suggesting that their dependence on others for the handling of economic affairs increased the risk of being exploited. Third, older adult people with impaired IADL were more prone to economic exploitation, with *p* < 0.001**. This indicated that they relied on others in handling economic affairs and increasing the risk of exploitation. The adequacy and maturity of family care was an important safeguard against financial exploitation. In addition, the risk of economic exploitation was higher among older persons in rural areas, with lower literacy and single economic source. To reduce the risk of economic exploitation, the following measures are recommended: Family and social support networks need to be strengthened. There is a need to improve ADL and provide assistance with financial matters. Family care needs to be enhanced, especially the maturity of family members in dealing with the older adult. In summary, numerous factors contribute to the prevalence of financial exploitation of the older adult, which must be avoided through multi-level interventions. The study provides an empirical basis for the development of strategies to prevent economic exploitation of older persons, which will help to ensure the economic security of older adults and improve their QOF and social well-being.

## Data Availability

The original contributions presented in the study are included in the article/supplementary material, further inquiries can be directed to the corresponding author.
